# Occurrence of microplastics in pellets from the common kingfisher (*Alcedo atthis*) along the Ticino River, North Italy

**DOI:** 10.1007/s11356-020-10163-x

**Published:** 2020-07-21

**Authors:** Anna Winkler, Alessandro Nessi, Diego Antonioli, Michele Laus, Nadia Santo, Marco Parolini, Paolo Tremolada

**Affiliations:** 1 Department of Environmental Science and Policy, University of Milan, Via Celoria 26, 20133 Milan, Italy; 2 University of Piemonte Orientale, DISIT, Via T. Michel 11, 15121 Alessandria, Italy; 3 Unitech NOLIMITS, Imaging facility, University of Milan, Via Golgi 19, 20133 Milan, Italy

**Keywords:** Microplastic, Common kingfisher, Freshwater ecosystem, μ-FTIR, SEM-EDS, Plastic ingestion, Ticino River

## Abstract

Previous research has reported avian plastic ingestion in marine bird species. Yet, while research attention on plastic pollution is shifting from marine to freshwater ecosystems, very few information on plastic ingestion is available for freshwater birds. Here, we examined the presence of microplastic in regurgitated pellets of the common kingfisher (*Alcedo atthis*) collected along the Ticino River (North Italy). In total, 133 kingfisher’s pellets were examined between March and October 2019 from 54 transects along the river. Plastic elements were detected and identified by visual inspection followed by μ-FTIR and SEM-EDS. Overall, we found 12 (micro)plastics from at least three different polymers in 7.5% of the pellets. This study provides the first report of plastic uptake of this bird species. It highlights the importance of spectroscopic techniques in plastic monitoring studies in order to avoid misidentification of items found. Documenting the presence of plastic ingestion by top carnivores such as fish-eating birds is necessary to understand the pervasiveness and impact of (micro)plastic pollution in food webs of freshwater ecosystems.

## Introduction

The interest in the study of microplastics (synthetic polymers < 5 mm in size; Arthur et al. 2009) in freshwater ecosystems is increasing continuously. The presence of microplastic in freshwater has been reported worldwide (Dris et al. 2018; Lahens et al. 2018; Mani et al. 2016, amongst others) and recently also in Italy (Campanale et al. 2019). Thus, it is not surprising that research attention is shifting towards the entire freshwater ecosystem and its food web, including fish-eating birds which are on the top level of this food web (Gochfeld et al. 1999). Several studies have investigated avian plastic ingestion in a wide array of marine species from offshore and coastal waters (e.g., Acampora et al. 2016; Avery-Gomm et al. 2013; Franco et al. 2019). However, information about (micro)plastic ingestion by continental birds is still scarce (Blettler et al. 2018; Reynolds and Ryan 2018). A recent study has pointed out the necessity of exploring the presence of (micro)plastics in waterbirds because this might represent a potential problem for continental freshwater bird conservation (Gil-Delgado et al. 2017).

Monitoring of plastic ingested by birds is mainly obtained by stomach analyses of dead birds (Acampora et al. 2014; Avery-Gomm et al. 2013; van Franeker et al. 2011). Methods for monitoring macro- and microplastics in living birds, however, are a suitable alternative for determining plastic contamination in the absence of finding dead animals. For bird species that do not accumulate plastic in their digestive tracts which can then be analysed in their faeces (Acampora et al. 2017a), the analysis of regurgitated pellets (indigestible residues of food composed of fish bones and teeth, scales, exoskeletons of insects, and other organic material) provides the information on the incidence of litter in the diet. One of the freshwater birds regurgitating pellets is the common kingfisher (*Alcedo atthis*, hereafter kingfisher). The species is distributed throughout most of Europe, Africa, and Asia and feeds on pelagic and benthic fish species and aquatic invertebrates (Čech and Čech 2015; Vilches et al. 2012). Their prey, mostly pelagic fish, is caught by a harpooning maneuvre of the kingfisher as he dives into the water, up to 25-cm deep (Fry et al. 2010). Plastic litter in regurgitates was analysed earlier in pellets from the great cormorant (Acampora et al. 2017a), in regurgitates from chicks of the black-legged kittiwake, northern fulmar, and great cormorant (Acampora et al. 2017b) in Ireland, and in pellets from Great Skua from the Farøe Islands (Hammer et al. 2016). However, these pellets were collected from marine species. There are currently no published data on plastic in pellets from freshwater birds, such as the kingfisher.

Therefore, the aim of this study was to assess the ingestion of (micro)plastic items by the kingfisher by analysing its regurgitated pellets collected along the Ticino River (North Italy). Hereby, we used an integrated approach relying on a preliminary visual inspection and the further application of two different analytical techniques, namely micro-Fourier transform infrared spectroscopy (μ-FTIR) and scanning electron microscopy coupled with energy-dispersive X-ray spectroscopy (SEM-EDS). Previous studies already stressed the importance of spectroscopic techniques in plastic monitoring studies as they are critical in avoiding misidentification of natural items for synthetic polymers (Nicastro et al. 2018; Wesch et al. 2016). In particular, when analysing small plastic items such as microplastic fibres which can be easily mistaken for cotton fibre and vice versa, thus, the objectives of the present study were (i) to provide information on the presence of plastic in pellets from a freshwater, fish-predatory bird; (ii) to highlight the necessity of using analytical methods for plastic identification and especially high-resolution techniques; and (iii) to investigate the spatial variation of pellets containing microplastics along the length of the Ticino River.

## Material and methods

### Sample collection

The sampling of a total of 133 kingfisher’s pellets was implemented between March and October 2019 from 15 out of 54 examined transects along the Ticino River (North Italy). The sampling transects were located along the main course and tributaries of the river with an average length of 551 ± 364 m from the Lake Maggiore down to the confluence into the Po River. The transects were examined for pellets under potential resting points of the kingfisher two times at a minimum (up to six times once pellets were detected in a transect). Visual observation of the kingfisher or response to a bird whistle indicated that the species was distributed along the whole watercourse, confirming previous findings of its high occurrence in the Ticino valley (Casale 2015). A map of the study area with the locations of pellets is shown in the result section.

### Sample preparation and microplastic extraction

In the laboratory, pellets were placed in glass petri dishes and visually inspected for the presence of potential plastic items (e.g., fragments, particles, fibres) under a stereo microscope (Wild M3B Heerbrugg Switzerland, 6.4 X – 40 X) with a detection and selection limit of 40 μm for particles and fragments. Most fibres had a thickness < 40 μm but were detected due to their length. Images were taken with the camera of the stereo microscope to measure the size of detected items using the imaging software Fiji. The putative plastic items were transferred into small glass vials with metal tweezers or needle and stored until analysis of their polymeric composition by μ-FTIR. The detection and identification limit with regard to particle size for the applied μ-FTIR instrument was 10 μm. Considering the detection and selection limit of the visual identification with the stereo microscope and the resolution limit of the μ-FTIR instrument, the application of an additional extraction method followed by an analytical high-resolution technique, which is the advantage of SEM-EDS (identification limit of 3 μm), was necessary in order to determine the occurrence of even smaller plastic items.

Therefore, after visual inspection and removal of the items for μ-FTIR analysis, the remaining pellet material was further processed for density separation extraction and SEM-EDS analysis. Out of all pellet material, we chose ten pellets from different transects representing the northern and southern areas of the Ticino River (five each). The material was placed in a beaker and underwent a density separation in 100 mL of a saturated sodium chloride solution (NaCl, 0.0616 M) for 24 h to settle parts of the prey such as bones and to float MP items. Subsequently, the supernatant was filtered with an in-house manufactured glass filtration apparatus on silver membrane filters (Sterlitech, 0.8-μm pore size, 13-mm diameter, filtration area of 19.6 mm^2^). The filters were dried for 48 h in a glass desiccator and stored until analysis with SEM-EDS.

### Quality control precautions

In order to assess potential sample contamination by atmospheric deposition of microplastic, we included procedural blanks into our analysis. In the field, an aluminium foil was placed next to a detected pellet and remained open for the duration of pellet collection, then closed, transported, and stored in the same way as the pellet samples. The blanks were taken for each sampling date and transect where a pellet was found. In the event that more than ten pellets were found in one transect of the same date, one blank per ten pellets was taken. In total, 29 blanks were sampled in the field. These field blanks were then further used to include potential airborne contamination of microplastic in the laboratory. For the duration of the visual analysis of pellets under the stereo microscope, field blank samples were opened and placed next to the microscope. All field blanks were then examined under the stereo microscope to search for potential deposited microplastics.

Additionally, for the extraction procedure as preparation for the SEM-EDS analysis, two blank samples deriving from the northern and southern project areas were processed and analysed in the same way as the pellet samples. All glassware were cleaned with washing detergent and rinsed with ultrapure Milli-Q water, which was previously filtered through cellulose membrane (0.45 μm). All the laboratory procedures were implemented on pre-cleaned surfaces, and executive persons wore cotton lab coats and washed their hands regularly.

### Sample analysis

FTIR microscopy was performed using a Thermo Scientific Nicolet iN10 MX Infrared Imaging Microscope. Items identified as putative plastics during the preliminary visual inspection by the stereo microscope were transferred individually on a silver membrane filter and placed onto the specimen holder of the instrument. Measurement of all particles was carried out in reflection mode in a wavenumber range of 4000–650 cm^−1^ controlled by OMNIC Picta software. A total of 256 scans were taken for each spectrum, with a spectral resolution of 4 cm^−1^. A detected polymer was considered as such when the match to the respective polymer in the library was > 70%.

For SEM-EDS analysis, high-resolution images of the filtrated sample material on the silver filter were taken. Polymer particles or fibres were identified by their elemental composition signatures. After blank and sample filters were attached to standard SEM stubs and coated with a thin film of evaporated gold, they were placed in a Zeiss LEO 1430 SEM coupled with an Oxford detector for EDS analysis. The analysed area/filter area ratio of the silver membrane filter was 11.46% (25 fields vertically, 20 fields horizontally, magnification × 500, total analysed area 2.25 mm^2^). Particles and fibres detected in the cross-shaped transect were analysed by EDS using the Oxford Instruments INCA ver. 4.04 software (Abingdon, UK). The smallest particle size/fibre thickness analysed was 3 μm (identification limit of the EDS). Operating conditions were the following: accelerating voltage 20 kV, probe current 80 μA, and working distance 15.0 mm. The expected elemental composition of polymers corresponds to their composition stoichiometry. For example, for polyethylene terephthalate (PET – (C_10_H_8_O_4_)_n_), the stoichiometric C:O ratio is 73:27, given that hydrogen does not provide a detectable peak in the EDS spectrum. Preoperational EDS tests on pure PET material confirmed the elemental ratio (with a gold trace of gold ~ 1%), with 5% variability for each element. A particle found on the filter was identified as PET when EDS spectra revealed the same elemental composition and when measured peaks matched those of spectra from the preoperational test. Elemental composition of 100% carbon indicated polyolefin composition, such as polyethylene (PE), polypropylene (PP), or polystyrene (PS), but could not be further specified since these polymers consist of no other elements besides carbon and hydrogen to distinguish them from each other. After the cross-shaped transect was analysed, the entire filter surface was scanned (× 30) looking for fibres and particles and inspected for their elemental composition. Potential confusion of synthetic fibres with natural textile fibres or fibres from drying paper tissue was prevented by measuring elemental composition of wool, cotton, and paper resulting in a mean ratio of C:O:N:S = 62:19:17:2, C:O = 61:39, and C:O = 66:34, respectively.

## Results

**Fig. 1:**
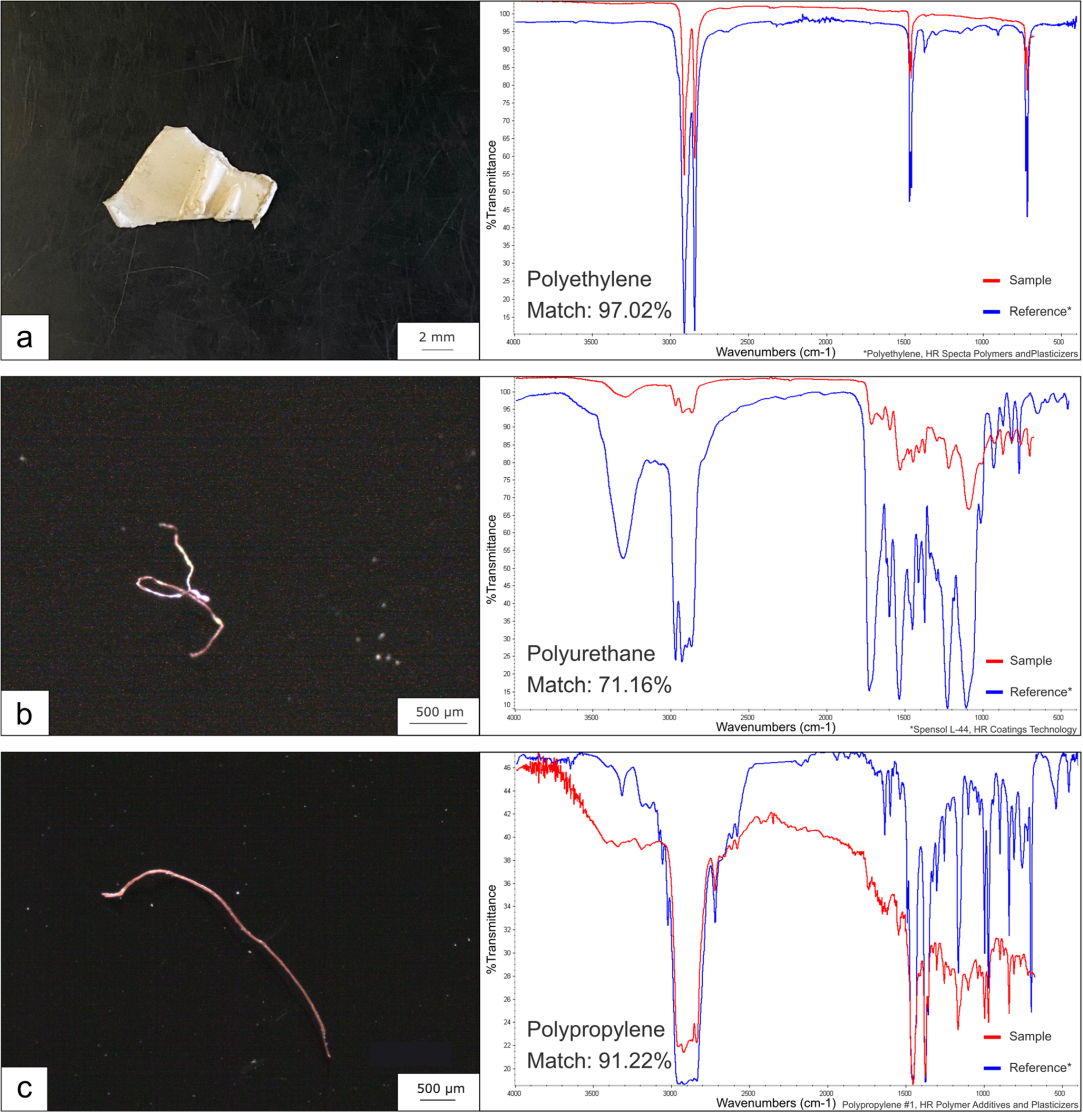
Microscope images of visually identified microplastics in pellets of the kingfisher and respective μ-FTIR spectra (red) with matches (%) to reference polymer from the library (blue). **a** Polyethylene fragment with a match of 97.02%. **b** Polyurethane fibre with a match of 71.16%. **c** Polypropylene fibre with a match of 91.22%

**Fig. 2:**
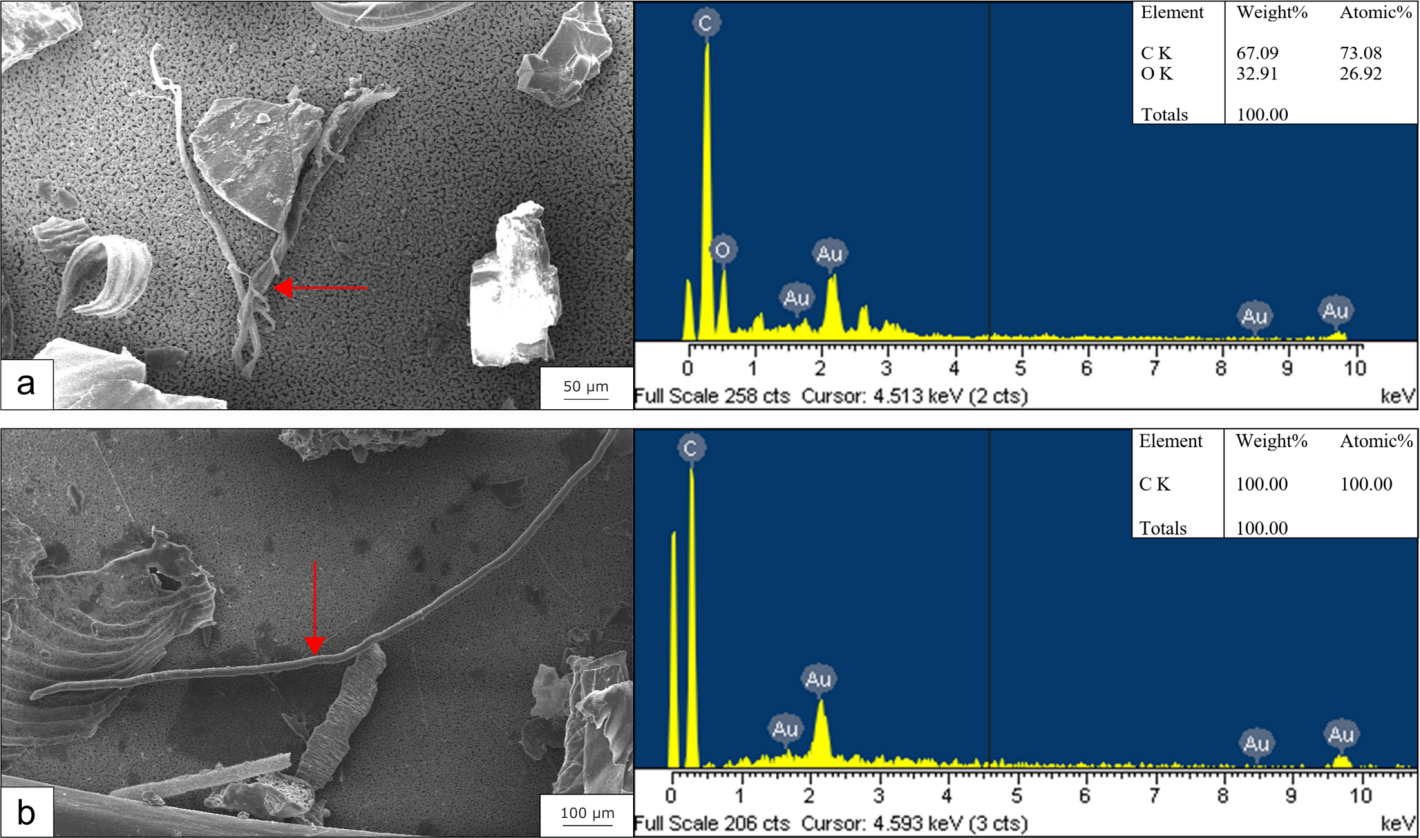
SEM images and respective EDS spectra with elemental quantitative data of analysed fibres from filtered extracts of pellets of the kingfisher. **a** Microplastic fibre on silver filter with specific elemental composition and C:O ratio corresponding to PET. **b** Microplastic fibre on silver filter with specific elemental composition of C = 100% indicating polymeric origin. Gold (Au) was removed from the output listing as it derived from the gold-coating

Overall, 12 plastic items were detected in 10 out of 133 regurgitated pellets from the kingfisher along the Ticino River. Micro-FTIR analysis of items detected under the stereo microscope determined three plastic elements in three different pellets (one from the northern, two from the southern project area). The identified polymers were a fragment of polyethylene (Fig. 1a), a polyurethane fibre (Fig. 1b), and polypropylene fibre (Fig. 1c). Elemental analysis by SEM-EDS performed on a subset from the pellet material (five pellets from the North and South, respectively) revealed five fibres with a C:O ratio corresponding to that of PET (Fig. 2a) and four fibres with the elemental composition of 100% carbon indicating polyolefin composition (Fig. 2b) from, in total seven different pellets (three from the North and four from the South). The detected microplastics were mainly fibres of very small size with a mean length of 1.16 ± 1.22-mm standard deviation, ranging from a length of 63 μm to 3.09 mm.

**Fig. 3:**
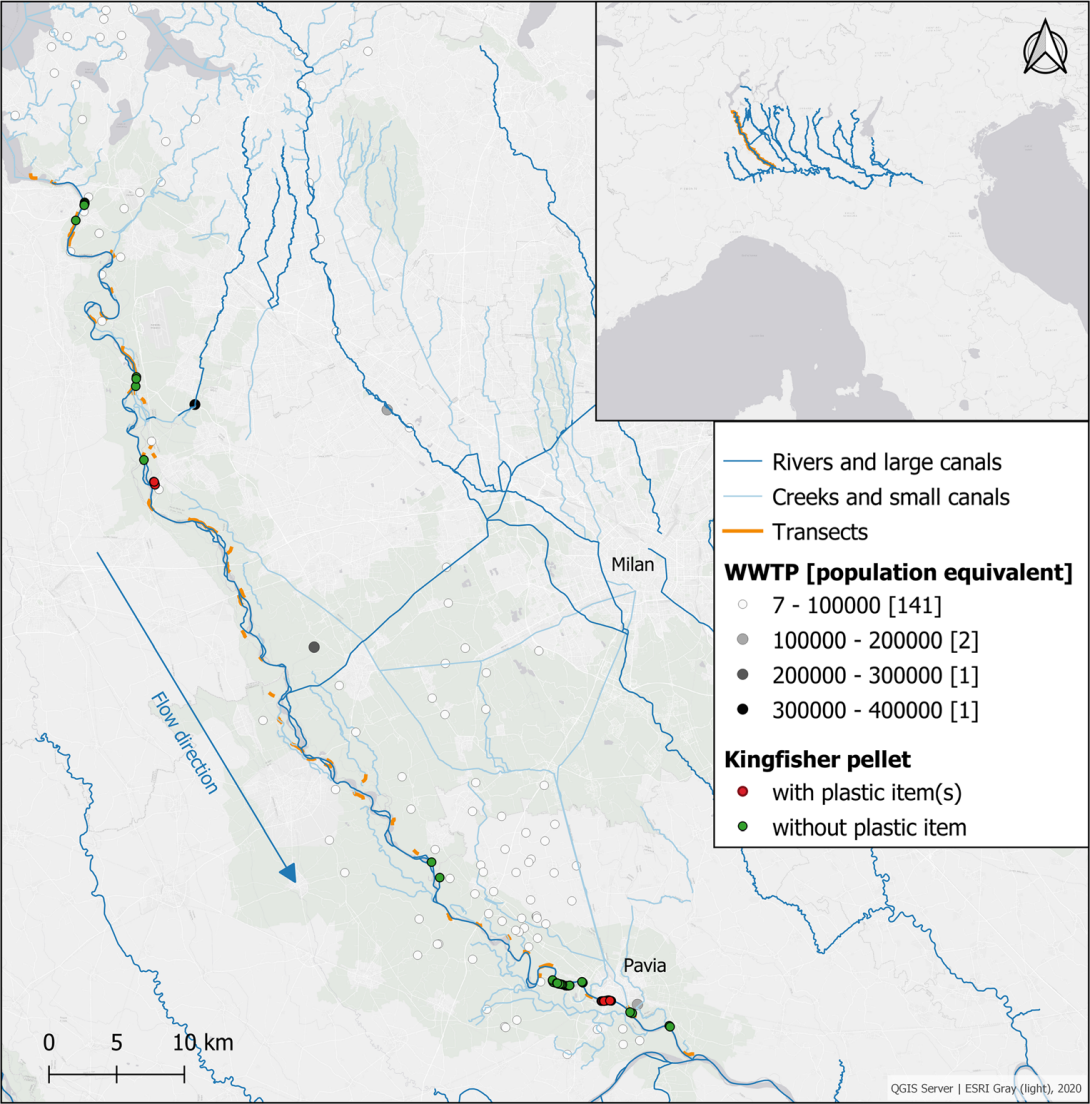
Map of the Ticino River with transects (orange lines) where the search for kingfisher pellets took place. Green spots represent pellets in which no plastic item could be identified by visual inspection and μ-FTIR analysis. Red spots represent pellets in which plastic items were determined by visual inspection and μ-FTIR or SEM-EDS analysis. Grey-scaled dots are wastewater treatment plants (WWTP) for different population equivalents

While the presence of the kingfisher was confirmed throughout the whole watercourse of the river (visual observations and response to bird whistle), most pellets (82%) were found in the southern area of the river. Considering the geographical distribution of pellets which included plastic items, no strong spatial variation (for example, an increase of microplastics in pellets over the length of the Ticino River) could be detected (Fig. 3). No plastics were detected in pellets in the top northern part of the study area. Following the watercourse, the first occurrence of plastic items in pellets was discovered downstream of a potential primary input source: a large wastewater treatment plant (WWTP) with a population equivalent > 300,000 that discards its effluent into the Ticino River (black dot in map of Fig. 3). The other transects, in which pellets with microplastic were found, were located in the southern part of the study area, where the river crosses the city of Pavia (~ 70,000 inhabitants). The urban area next to the riverside represents a large (micro)plastic source by means of water runoff or atmospheric deposition.

Procedural field blanks were examined under the stereo microscope, but no putative microplastics were found that could have been analysed by μ-FTIR. Moreover, no microplastic fibres were detected in the two procedural blanks analysed by SEM-EDS; hence, airborne contamination during sampling in the field and analysis in the lab was efficiently minimised.

## Discussion

In this study, 7.5% of pellets (10 out of 133) were contaminated by microplastics. Out of the 12 detected microplastics, 11 were fibres. Fibres are often reported as the most abundant microplastic items in water (Campanale et al. 2019; Kay et al. 2018; Mani et al. 2016, amongst others) but also in fish (Collard et al. 2015). Dris et al. (2018) reported that fibres often represent the most common microplastics in surface water of rivers, mainly when sampling of water with a mesh size lower than 300 μm was applied. A key source of fibres is from the breakdown of textiles in washing machines entering the river through the effluent of wastewater treatment plants (Kay et al. 2018). Consequently, it is not surprising that fibres were the predominant form of microplastic found in digestive tracts of fish, such as chubs (*Squalius* spp.) (Collard et al. 2015), which is one of the main genus (in juvenile stage) composing the diet of the kingfisher from the Ticino River (Nessi 2020). In general, juvenile stages of the predominant fish species in the diet of the Kingfisher, such as chubs and barbels (*Barbus* spp.), are exposed to fibres, as they inhabit shallow areas close to the water surface where fibres are floating. However, McGoran et al. (2017) found microplastic fibres also in benthic fish species, indicating that not only fish from shallow habitats ingest fibres. Microplastic fibres in the Ticino River were already detected before, in faeces from otters, a mammal top predator who also feeds on chubs and barbels (Smiroldo et al. 2019). As shown in Fig. 3, the Ticino River receives the effluents of several WWTPs, canals, and smaller rivers, draining also residential, commercial, and industrial areas to the west of the city of Milan. All these discharges are potential input sources of microplastics to the river. Thus, it can be assumed that the microplastic load of the river water increases with its length, as also proven in other studies (Eerkes-Medrano et al. 2015; Mani et al. 2016). The amount of microplastic in pellets from the kingfisher did not reflect this presumed spatial variation; however, it can be related to specific sources as reported in the “Results” section.

### Sources of microplastic to the kingfisher

Unlike birds which feed at the water surface, diving birds such as the kingfisher are less prone to directly ingest floating plastic material from the water due to the buoyant nature of most plastic items. Considering that diving birds take up plastics more likely through their prey by secondary ingestion, as reported by Acampora and co-authors (2017a), also, the largest detected fragment in the present study (polyethylene of 12.1 mm on its longest side, Fig. 1a) could also be ingested secondarily. Although microplastics can be accidentally ingested from water during fish predation, the possibility that fish already ingested microplastics is high. Therefore, we can speculate that particles and fibres found in kingfisher pellets derive from the diet, and that microplastic was transferred through the food web. Since the regurgitation of pellets presents an effective method of ejecting plastic items, the physical effects are considered to be lower than for non-regurgitating bird species. However, the toxicological consequences of plastic ingestion by birds caused by related chemicals such as additives and micropollutants attached to their surface are poorly studied. Toxins leaching from the plastics upon ingestion are considered to induce adverse health effects in birds, including endocrine disruption and reduced reproduction (Giesy et al. 2003; Roman et al. 2019).

### Comparison with other freshwater birds

Only few previous studies showed the ingestion of plastics by freshwater birds. Plastic (mainly fragments) was detected in 9% (*n* = 148; English et al. 2015) and 11% (*n* = 350; Holland et al. 2016) of digestive tracts of ducks and geese in Canada. In South Africa, 5% of analysed faeces (*n* = 283) from different ducks and goose species were found to contain microplastic in the form of fibres (Reynolds and Ryan 2018). Although our findings (7.5% of analysed pellets contained microplastic, *n* = 133) fall into the range of these previous results, a comparison of our findings with the results of the studies mentioned above is difficult, as they investigated big-sized, mainly herbivorous bird species. For these species, microplastic ingestion derives most likely from sediment particles and water than from their food, although it is not proven. On the contrary, here, we prove microplastic ingestion from a top predator of river ecosystems, for which the ingestion more likely derived from their food rather than from abiotic elements (sediment and water). Moreover, these previous studies did not apply spectroscopic or elemental techniques for identification of smaller microplastics, which was the purpose of the present work.

### Methodological novelties and issues

This study is the first to investigate microplastic load in pellets of a freshwater bird. The kingfisher regurgitates a pellet a few times per day (Fry et al. 2010). Therefore, the sampled pellets may represent only the last ingested meal(s) consumed throughout the same day. Hence, the amount of plastic in the pellets reflects the ingestion over a short-time period, as items could have been previously expelled. Although regurgitated pellets are easily collected, we are aware that the samples may show varying levels of digestion depending on the duration between prey ingestion and final pellet ejection. This aspect might also influence instrumental plastic detection.

Apart from these considerations, another crucial element should be pointed out: unlike the studies mentioned above, in this work, two complementary techniques were applied, μ-FTIR and SEM-EDS. In fact, out of the 12 detected microplastics, three were identified by stereo microscope inspection and μ-FTIR analysis and nine by an additional density separation extraction and high-resolution SEM-EDS analysis. The confirmation by analytical techniques is essential, as natural fibres from wool or cotton can be mistaken for synthetic ones. In fact, from our visual analysis under the stereomicroscope, a high number of items were selected as possible microplastics, of which only three were confirmed as such by μ-FTIR. During the SEM-EDS analysis, many particles with fibre and non-fibre shapes were analysed, yet only nine fibres were confirmed as microplastics. In the case of non-fibre particles, some of them were compatible with the elemental composition of plastic polymers, but particles with similar shape and elemental composition were found in blanks (even if in lower abundance) so that we decided to not consider non-fibre small particles as potential microplastics in the pellet samples. As stated before, polymer identification by SEM-EDS is limited to polymers which consist of other elements besides carbon and hydrogen, such as oxygen in polyethylene terephthalate (PET) and chlorine in polyvinyl chloride (PVC). Thus, SEM-EDS is complementary rather than a substituting technique for other analytical methods such as μ-FTIR.

Regarding the instrumental confirmation of the microplastic detection of this study and the precaution criteria used for determining their presence, the reported findings can underestimate the real number of microplastics in the kingfisher diet. Especially considering that all the pellets were inspected visually under the stereo microscope for analysis by μ-FTIR, only a subset of samples was analysed by density separation and SEM-EDS analysis for smaller particle detection. This part of the research was added mainly for comparative methodological purpose.

## Conclusions

This study is the first assessment of the abundance of (micro)plastics in pellets of the common kingfisher. All (micro)plastics were fibres except one larger fragment. Rivers flowing through highly urbanised regions, like the Ticino River, are particularly vulnerable to plastic input, primarily through the effluent of wastewater treatment plants. Indeed, although the Ticino River is included amongst the global biosphere reserve network and is protected by two regional parks, it is subject to considerable plastic contamination. The present findings of plastics in kingfisher pellets confirmed this condition, although plastic contamination of pellets does not reflect assumed increased microplastic load over the length of the river. The kingfisher, being a top-level predator, takes up plastic particles and fibres most likely through secondary ingestion. It seems that the regurgitation of indigestible material is an effective method for the ejection of the ingested plastic particles. Thus, the kingfisher has an advantage over those species which accumulate plastic in their stomach.

The use of regurgitated pellets and the analysis with μ-FTIR and SEM-EDS proved to be a suitable method to assess plastic exposure for freshwater birds like the kingfisher. The limits of SEM-EDS polymer identification to only polymers consisting of other elements than just carbon make this rather a complementary technique than a substitute for μ-FTIR. Nevertheless, by applying high-resolution imaging by SEM-EDS, we confirmed the prevalence of ingested fibres that are not easily detectable under stereo or binocular microscopes. Thus, the results of this work indicate a strong underestimation of microplastic pollution in freshwater birds when only visual inspection under the stereo microscope is applied. Therefore, we suggest the tied use of different analytical techniques for identifying (micro)plastics in pellets and, more general, in future avian plastic ingestion studies, as we observed fibres by SEM-EDS that we did not detect earlier via visual inspection under the stereo microscope.

This study on microplastic in regurgitated pellets presents a baseline for future studies that should be carried out to further assess microplastic ingestion rates for freshwater birds. Plastic presence/absence studies through the collection of pellets are minimally invasive and provide insights into the plastic exposure of freshwater birds that are alive. Several features make the kingfisher an excellent monitoring species: it is widely distributed, site-loyal, and obtains all its food from aquatic systems at a high feeding rate. Moreover, its small size can be considered as a peculiar characteristic making it more suitable for small size microplastic monitoring than bigger birds. Reporting plastic ingestion by top carnivores such as fish-eating birds is necessary to understand the pervasiveness and impacts of microplastic pollution in food webs of freshwater ecosystems. However, we highlight that the application of spectroscopic analytic techniques is crucial to allow for discrimination between plastic and natural items.

## Abbreviations

EDS: Energy-dispersive X-ray spectroscopy
μ-FTIR: Micro-Fourier transform infrared spectroscopy
PE: Polyethylene
PET: Polyethylene terephthalate
PP: Polypropylene
PS: Polystyrene
PVC: Polyvinyl chloride
SEM: Scanning electron microscopy
